# Supplementary material for: Congenital Zika Virus Infection Impairs Corpus Callosum Development
Source: Viruses. 2023 Nov 28;15(12):2336. doi: 10.3390/v15122336 (PMC10748342; doi:10.3390/v15122336)
Supplement: Supplementary file 1 [file viruses-15-02336-s001.zip › viruses-2702982-supplementary.pdf]

viruses

MDPI

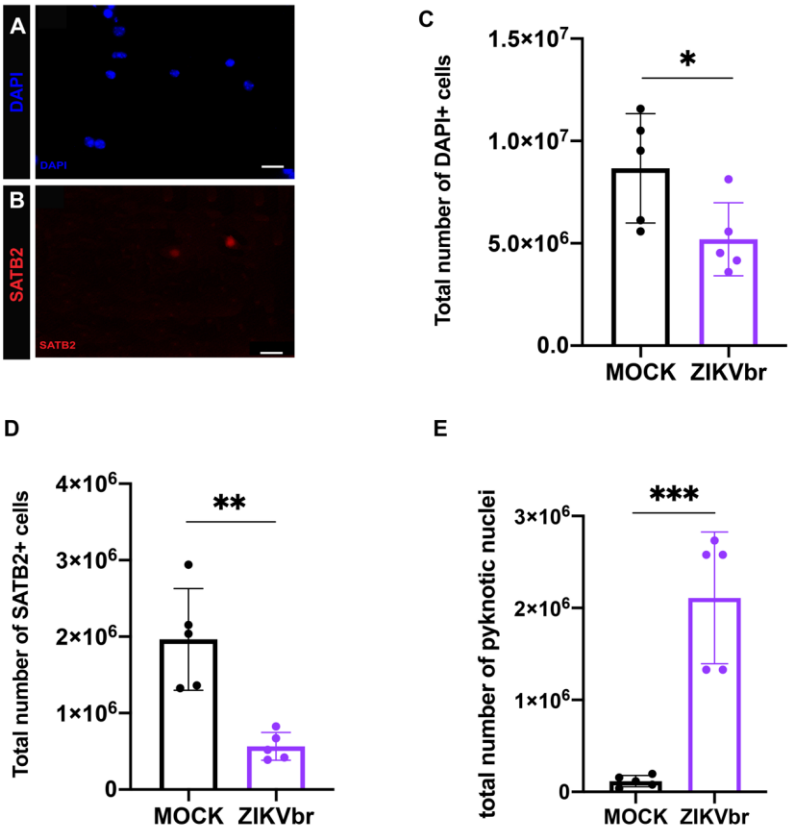

Supplementary Figure S1. Zika virus congenital infection at E15 reduces the total number of callosal neurons and increases pycnotic nuclei at P4. (A, B) Cortical brain nuclei labeling with (A) DAPI (blue) and (B) SATB2 (red) after Isotropic fractionator technique. Scale bars = 10μm (C) Quantitative analysis for cortical absolute number of cells (DAPI staining in blue) in infected and control tissue. (Unaired t-test, t=2,413, df=8, p= 0,0423) N= MOCK (5) ZIKV (5). (D) Total number of callosal neu-rons - SATB2 staining (red) on cell nuclei isolated with the isotropic fractionator technique in ZIKV and MOCK animals. (Unaired t-test, t=4,542, df=8, p= 0,0019) N= MOCK (5) ZIKV (5)(E) Quantifica-tion of pyknotic nuclei using the isotropic fractionator technique. (Unaired t-test, t=6,198, df=8, p= 0,0003) N= MOCK (5) ZIKV (5). $^*p \leq 0.05$, $^{**}p \leq 0.01$, $^{***}p \leq 0.001$
